# Is laparoscopic colorectal cancer surgery associated with an increased risk in obese patients? A retrospective study from China

**DOI:** 10.1186/1477-7819-12-184

**Published:** 2014-06-11

**Authors:** Xiang Xia, Chen Huang, Tao Jiang, Gang Cen, Jun Cao, Kejian Huang, Zhengjun Qiu

**Affiliations:** 1 Department of General Surgery, Shanghai Jiaotong University Affiliated First People’s Hospital, 100 Hai Ning Road Shanghai 200080 People’s Republic of China

**Keywords:** Complication grading system, Laparoscopic colorectomy, Obesity patients

## Abstract

**Background:**

The impact of obesity on surgical outcomes after laparoscopic colorectal cancer resection in Chinese patients is still unclear.

**Methods:**

We retrospectively reviewed the prospectively collected data from 527 consecutive colorectal cancer patients who under went laparoscopic resection from January 2008 to September 2013. Patients were categorized into three groups: nonobese (body mass index (BMI) <25.0 kg/m^2^), obese I (BMI 25.0 = to 29.9 kg/m^2^) and obese II (BMI ≥30.0 kg/m^2^). Clinical characteristics, surgical outcomes and postoperative complications were compared between nonobese, obese I and obese II patients.

**Results:**

From among the 527 patients, there were 371 patients with in the nonobese group, 142 patients in the obese I group and 14 patients in the obese II group. The patients were well-matched for age, sex and American Society of Anesthesiologists class, except for BMI (*P* = 0.001). The median operative time correlated highly significantly with increasing weight (median: nonobese = 135 minutes, obese I = 145 minutes, obese II = 162.5 minutes; *P* = 0.001). There appeared to be a slight tendency toward grade III complications (rated according to the Clavien-Dindo Classification of Surgical Complications) in the obese II group, but this difference was not significant (nonobese = 5.1%, obese I = 3.5% and obese II = 14.3%; *P* = 0.178). None of the grade III complications which occurred in the obese II group were wound dehiscences that required a stitch. Other aspects, such as estimated blood loss, harvested lymph nodes, operation type, pathological results, conversion rate and overall postoperative complications, were not statistically significant.

**Conclusion:**

With sufficient experience, laparoscopic colorectal cancer surgery is feasible and safe in obese Chinese patients.

## Background

Obesity has become a serious public health problem in both developed and developing countries [1]. In China, the current population is characterized by a growing prevalence of obesity (26.9% in men and 31.1% in women). *Overweight *is defined as a body mass index (BMI) of 25 kg/m^2^ or greater [2]. BMI is the most practical method (and also the standard method) of assessing total body fat. In some studies, researchers have found that patients with high BMI values are more technically demanding to treat and have a higher risk of perioperative complications [3-5].

Surgical techniques and skills improved magnificently in recent decades, and laparoscopic surgery has gradually become a widely accepted approach in the treatment of patients with colorectal cancer [6,7]. For obese patients, some surgeons have consistently regarded obesity as a relatively significant contraindication to laparoscopic colorectal resection [8-10]. In recent studies, investigators have shown that laparoscopic colorectal cancer surgery was feasible and safe in obese patients treated at some hospitals with experienced surgeons [11-14], despite the increased conversion rate, operation duration or longer hospital stay [14-16]. Many of these articles describe the impact of overweight on colorectal cancer patients undergoing laparoscopic surgery in Western countries. Few clinical data or studies have been reported regarding patients in China. The purpose of our study was to evaluate the feasibility and safety of laparoscopic colorectal cancer resection in obese Chinese patients.

## Methods

### Ethical approval

This retrospective study was approved by the Clinical Trial Ethics Committee of Shanghai Jiaotong University Affiliated First People’s Hospital. All patients provided their written informed consent.

### Database

From January 2008 to September 2013, all consecutive colorectal cancer patients who were treated by laparoscopic colorectal excision in Shanghai Jiaotong University Affiliated First People’s Hospital were included in a prospective database. Exclusion criteria for our study were synchronous metastasis, conversion to open surgery because of intraoperative events, missing any necessary data (such as height or weight), any combined resection, and not an R0 resection. Surgery was performed in all patients by one very expert surgeon (ZQ, the chief of the general surgery department at Shanghai Jiaotong University Affiliated First People’s Hospital). The selection criteria for patients who underwent laparoscopic surgery did not change throughout the study period.

The patients’ preoperative demographic and clinical characteristics included age, sex, BMI and American Society of Anesthesiologists class. The surgical data we gathered consisted of operative time, estimated blood loss, harvested lymph nodes, operation type and pathological results. Postoperative complications, urinary catheter out time, start time of fluid intake and length of postoperative hospitalization were considered in assessing postoperative outcomes. In accordance with the proposed International Obesity Task Force classification system, we used the following BMI cut off points to assign each patient to one of three groups: nonobese (BMI <25.0 kg/m^2^), obese I (BMI 25.0 to 29.9 kg/m^2^) and obese II (BMI ≥30.0 kg/m^2^). Surgical complications were defined according to the Clavien-Dindo Classification of Surgical Complications as grades I, II, III, IV and V [17]. The precise preoperative preparations, operation procedures and postoperative management have been described previously in detail [18]. Identical criteria were used for each group in terms of preoperative preparation, postoperative management and discharge to home.

The data are presented as mean ± SD (parametric data) or median ± range (nonparametric data). The data were compared using Student’s *t*-test for paired variables, the Mann–Whitney *U *test for unpaired continuous variables and χ^2^ or Fisher’s exact test for discrete variables. The SPSS 13.0 statistical software package was used for statistical analysis (SPSS Inc, Chicago, IL, USA). A *P*-value <0.05 was considered significant.

## Results

The classification of 527 patients according to BMI is presented in Table 1. The mean BMI of the entire patient cohort was 23.0 ± 3.2 kg/m^2^. From among the 527 total patients, 371 were in the nonobese group, 142 were in the obese I group and 14 were in the obese II group. The mean BMI in the obese II group was significantly higher than those of the nonobese group (*P* = 0.001) and obese I group (*P* = 0.001). In addition, comparison of these groups did not reveal a highly significant increase in the conversion rate (nonobese: 2.6%, obese I: 2.7%, obese II: 12.5%; *P* = 0.113). Aside from BMI, there was no significant difference between the patients in the three groups.

**Table 1:** **Clinical characteristics of thepatients**^**a**^

**Characteristics**	**Nonobese (<25.0 kg/m**^**2**^**) ( *N *** **= 371)**	**Obese I (25.0 to29.9 kg/m**^**2**^**) (*N *** **= 142)**	**Obese II (≥30 kg/m**^**2**^**) (*N *** **= 14)**	***P*-value**
**Mean age , yr**	68.6	69.7	72.5	0.109
**Males, *n *** **(%)**	185(49.9%)	78(54.9%)	8(57.1%)	0.537
**Mean BMI, kg/m**^**2**^	21.5	26.6	30.9	0.001
**ASA class, *n *** **(%)**				0.295
**I/II**	310(83.6%)	112(78.9%)	10(71.4%)	
**III**	61(16.4%)	30(21.1%)	4(28.6%)	
**Comorbidity**				0.589
**Yes**	71(19.1%)	22(15.5%)	2(14.3%)	
**No**	300(80.9%)	140(84.5%)	12(85.7%)	
**Operation type, *n *** **(%)**				0.963
**Right colectomy**	123(33.2%)	47(33.2%)	5(35.7%)	
**Left colectomy/sigmoidectomy**	198(53.4%)	73(51.4%)	8(57.1%)	
**Low anterior resection**	20(5.4%)	11(7.7%)	0(0%)	
**Abdominoperineal resection**	30(8.0%)	11(7.7%)	1(7.2%)	
**pT stage, *n *** **(%)**				0.861
**T1**	42(11.3%)	16(11.3%)	1(7.1%)	
**T2**	62(16.7%)	27(19.0%)	1(7.1%)	
**T3 orT4**	267(72.0%)	99(69.7%)	12(85.7%)	
**Conversion**^b^, *n* (%)	10(2.6%)	4(2.7%)	2(12.5%)	0.113

^a^ASA, American Society of Anesthesiologists; pT, Primary tumor. ^b^These 16 conversion patients were presented alone, not counted in the other characteristics analysis.

Table 2 shows the surgical outcomes in each group. The median operative time correlated highly significantly with increasing weight (median: nonobese = 135 minutes, obese I = 145 minutes, obese II = 162.5 minutes; *P* = 0.001). In contrast, no significant inter group differences were found in terms of estimated blood loss and harvested lymph nodes.

**Table 2:** Surgical outcomes in nonobese, obese I and obese II groups

	**Nonobese (<25.0 kg/m**^**2**^**) ( *N *** **= 371)**	**Obese I (25.0 to 29.9 kg/m**^**2**^**) ( *N *** **= 142)**	**Obese II (≥30 kg/m**^**2**^**) ( *N *** **= 14)**	***P*-value**
**Operative time,min**	135 (70 to 300)	145 (85 to 360)	162.5 (115 to280)	0.001
**Estimated blood loss,ml**	100 (10 to 750)	100 (20 to 750)	100 (50 to 600)	0.076
**Harvested lymph nodes, *n***	13 (1 to 79)	12 (1 to 36)	15 (4to 26)	0.111

Data are presented as median (range).

As summarized in Table 3, no significant differences were found between nonobese, obese I and obese II patients with regard to overall postoperative complications, mortality, urinary catheter out time, start time of fluid intake and length of post surgery hospitalization. It is noteworthy that the rates of postoperative grades I, II, III, IV and V complications were similar in these three groups (rated according to the Clavien-Dindo Classification of Surgical Complications). In subgroup analysis, there appeared to be a slight tendency toward grade III complications (surgical intervention) in the obese II group compared with the nonobese and obese I groups (nonobese: 5.1%, obese I: 3.5% and obese II: 14.3%; *P* = 0.178).

**Table 3:** **Patient postoperative course data**^**a**^

	**Nonobese (<25.0 kg/m**^**2**^**) ( *N *** **= 371)**	**Obese I (25.0 to29.9 kg/m**^**2**^**) ( *N *** **= 142)**	**Obese II (≥30 kg/m**^**2**^**) ( *N *** **= 14)**	***P*-value**
**Overall postoperative complications, *n *** **(%)**	112(30.2%)	46(32.4%)	6(42.9%)	0.561
**Total grade I, *n *** **(%)**	18(4.9%)	8(5.6%)	0	0.533
**Wound infection, *n***	13	5	0	
**Wound dehiscence, *n***	5	3	0	
**Total grade II, *n *** **(%)**	72(19.4%)	30(21.1%)	4(28.6%)	0.661
**Pyrexia of unknown origin, *n***	17	6	1	
**Urinary infection, *n***	6	5	1	
**Septicemia, *n***	2	1	0	
**Cerebral infarction, *n***	1	0	0	
**Postoperative hypertension, *n***	4	1	0	
**Anastomotic leak (treated conservatively), *n***	11	4	0	
**Pneumonia, *n***	4	1	0	
**Ileus (treated conservatively), *n***	7	5	1	
**Chyle leakage, *n***	2	2	0	
**Deep vein thrombosis, *n***	2	0	0	
**Atelectasis, *n***	1	0	0	
**Delirium, *n***	2	0	0	
**Urinary retention, *n***	6	2	0	
**Arrhythmia, *n***	3	0	0	
**Neutropenia, *n***	1	0	0	
**Intraperitoneal hemorrhage (necessitating transfusion), *n***	2	1	0	
**Respiratory infection, *n***	1	2	1	
**Total grade III, *n *** **(%)**	19(5.1%)	5(3.5%)	2(14.3%)	0.178
**Wound dehiscence (necessitating stitch), *n***	5	3	2	
**Pneumothorax, *n***	1	0	0	
**Anastomotic bleeding (endoscopic treatment), *n***	5	0	0	
**Anastomotic leak (necessitating reoperation), *n***	5	1	0	
**Anastomotic ischemia (necessitating reoperation), *n***	2	0	0	
**Intraperitoneal hemorrhage (necessitating reoperation), *n***	1	1	0	
**Total grade IV, *n *** **(%)**	2(0.5%)	2(1.4%)	0	0.378
**Respiratory failure, *n***	2	1	0	
**Cardiac failure, *n***	0	1	0	
**Total grade V, *n *** **(%)**	1(0.3%)	1(0.7%)	0	0.505
**Urinary catheter out**^**b**^**, days**	3(0 to 52)	4(1 to 21)	4(1–14)	0.269
**Start fluid intake**^**b**^**, days**	3(1 to 47)	3(1 to 20)	3 .5(1–10)	0.774
**Length of postoperative hospitalization**^**b**^**, days**	10(3 to 102)	11(6 to 84)	11.5(7–33)	0.353

^a^Anastomotic leak: Discharge of colon or rectum content via the drain, wound or abnormal orifice; Grade: Graded according to the Clavien-Dindo Classification of Surgical Complications; Ileus: Need for a nasogastric tube because of postoperative nausea, vomiting and abdominal distention or delayed oral intake for more than 5 days after surgery; Pyrexia of unknown origin: Any temperature above 37°C for more than 24 hours occurring after original pyrexia following surgery settled, for which no obvious cause was found; Respiratory failure: Respiratory difficulty requiring emergent ventilation; Septicemia: Positive blood culture; Urinary infection: Presence of >10^5^ bacteria/ml with the presence of white cells in the urine after previously clear urine; Wound dehiscence: Superficial or deep wound breakdown; Wound infection: Wound cellulitis or discharge of purulent exudates. ^b^Data are presented as median (range) unless otherwise indicated.

## Discussion

Colorectal cancer in obese patients is increasingly encountered in laparoscopic surgical practice. In theory, laparoscopic colorectal resection in obese patients is technically more demanding than in nonobese patients [19]. In some large randomized trials in which investigators evaluated open versus laparoscopic colectomy, obesity was either an exclusion criterion [20] or a main reason for conversion [21]. Moreover, obesity is considered to be associated with raised postoperative complications, including systemic failure or ileus, that lead to longer hospitalization [14,22]. Conversely, in some specialized centers, researchers have found that laparoscopic colectomy could be performed in obese patients with acceptable postoperative morbidity [16] and a shorter recovery [23,24]. The true relationship between obesity and laparoscopy is still heatedly disputed.

To the best of our knowledge, our present study is the first to evaluate the effect of BMI on short-term outcomes of Chinese colorectal cancer patients undergoing laparoscopic resection using the accurate classification of surgical complications proposed by Dindo *et al*. [17]. We found that operative time associated with laparoscopic resection was significantly increased in our obese I and obese II groups compared to the nonobese group (Table 2). Other relevant surgical data, such as estimated blood loss, conversion rate and harvested lymph nodes were not significantly different among these three groups. The significantly longer median operative times in the obese II group (27.5 minutes) and the obese I group (17.5 minutes) were of no practical importance, but had sufficient benefits in the context of laparoscopic surgery. This result is in accord with the data reported by Poulsen *et al*. [25]. The findings derived from our comparisons of these operative times are similar to those in other studies in which authors reported significant differences in the duration of surgery between obese and nonobese patients. Recently, in the largest study to date, Rheidbach *et al*. [26] found that operative time was longer for obese patients among a total of 5,853 colorectal cancer patients undergoing laparoscopic resection (obesity defined as BMI ≥30 kg/m^2^). In a Korean study (*N* = 984 consecutive patients who under went laparoscopic surgery for colorectal cancer) in which the researchers used the same definition of *obese*as we used in our present study, obese II and obese I patients had longer operative times than nonobese patients [13]. This significantly longer operative time occurs primarily because of the increased amount of fat in the mesentery, leading to a requirement for a more skilled mobilization of the intestines, colon and rectum during surgery. Furthermore, the identification of the vital artery and vein in the mesentery is more difficult. In other, previous studies, estimated blood loss was not higher in patients with BMI values from 25.0 to 29.9 kg/m^2^ or ≥30 kg/m^2^, revealing that laparoscopic colorectal resection in obese patients is merely more time-consuming than it is in nonobese patients, without a tendency to require transfusions [14,26,27]. Park *et al*., in their large cohort study, found that the estimated blood loss in obese II patients (BMI ≥30 kg/m^2^) to be significantly higher than that in Nonobese patients, but not than the blood loss in obese I patients [13]. With regard to conversion rate, in contrast to previous reports from Western countries which have shown that a BMI ≥30 kg/m^2^ had a negative impact on conversion rates [8,16], we did not find any significant difference in our study. This result, which derives from the fact that our stable surgical team has had a great deal of practice and experience which has proved to lower conversion rates [28,29], is consistent with the findings reported by Schwander *et al*. [27] and Leroy *et al*. [24].

Previously, in some studies done in Western countries, researchers have investigated the impact of obesity on the postoperative complications of laparoscopic colorectal surgery, whose results have made it, so far, a controversial matter [15,25,26]. We found a slightly higher risk of overall postoperative complications and longer hospitalization in the obese II group, but none of the differences achieved statistical significance. In the subgroup analysis, grade III complications in the obese II group were higher than in the other two groups, but with no statistical significance (14.3% versus 5.1% in the nonobese group and 3.5% in the obese I group) (Table 3). However, the two grade III complications that occurred in obese II group were wound dehiscences that necessitated stitching with local anesthesia. There were no incidences of anastomotic leak or anastomotic bleeding in the obese II group. Surgical therapy was a more common treatment used in obese II patients in cases where a wound infection or dehiscence occurred [27]. Park *et al*., who applied BMI criteria identical to those we used in our present study, stated that postoperative complications were similar between nonobese, obese I and obese II patients, but obese II patients had a significantly longer postoperative hospital stays. However, they concluded that laparoscopic surgery for colorectal cancer patients is feasible and safe, but requires special postoperative care [13]. This longer hospitalization phenomenon was not present in our study. These different postoperative findings between the two studies might result from Park *et al*.’s calculation of all surgical patients’ hospitalizations in their study, thus including conversion patients who would contribute to the longer hospital stays in their study. Furthermore, the explanation of these differing results might lie in the different experience levels of the surgeons involved [14]. In sharp contrast to Park *et al*., to lower selection bias, we excluded from our analysis conversion patients and several patients who under went surgery performed by less experienced surgeons.

BMI is a commonly used objective measure of body fat. On the basis of the definition of *obesity* set by the World Health Organization (WHO) (BMI ≥30 kg/m^2^), the rate of obesity in China is much lower than that in Korea. Because of this in appropriate cut off point for Asian populations, the International Obesity Task Force has proposed a special cutoff point for this demographic group [30]. Therefore, we utilized the WHO criterion to categorize our patients as nonobese, obese I or obese II. Despite some Japanese studies about the impact of obesity in which investigators found that visceral obesity is more useful than BMI in predicting surgical outcomes after laparoscopic colorectal surgery [31,32], BMI is still a cheaper and more practical tool to use in estimating a patient’s body fat, especially in a developing country such as China.

The most important limitation in our study is that it was non randomized and retrospective. Nonetheless, we report the first laparoscopic surgical outcomes comparison among obese and nonobese Chinese colorectal cancer patients. What’s more, most authors have classified these patients’ postoperative complications as either minor or major complications rather than using five grades as we did (according to the Clavien-Dindo Classification of Surgical Complications). In the future, we will design a prospective randomized control trial. Our data derive from operations performed by a single experienced surgeon. We made this choice to help us avoid any interpersonal variability made by two or more surgeons as well as any bias associated with the earlier stage of the learning curve. In addition, the number of patients in the obese II group was small. Because of racial disparities between Asians and Caucasians, the obese I group was more representative of Asians in general.

Keeping in mind these few limitations of our study resulting from its non randomized and retrospective design, we can still state that we found there were longer operative times in the obese I obese II groups than in the nonobese group, but no significant differences in postoperative complications between the nonobese, obese I and obese II patients. These results show that laparoscopic colectomy is indeed more technically demanding in obese than in nonobese patients. Also, we found that postoperative management required more meticulous care in the obese II patients because of the high possibility of wound dehiscence requiring stitches, in spite of the fact that overall postoperative complications did not differ significantly between the three groups. However, the unfavorable impact of obesity on surgical outcomes cannot become a contraindication to laparoscopic surgery in obese patients. This assertion is supported by the findings in a large cohort of study in which the researchers reported that the incidence of postoperative complications after elective general surgery did not differ between obese and nonobese patients [33]. As surgeons gain more experience in laparoscopic colorectal resection, this approach will become feasible and efficient in both obese and nonobese patients [27].

## Conclusion

In this study of patients who under went laparoscopic colorectal surgery performed by a surgeon with sufficient experience (thereby excluding from analysis the early stage of the learning curve), we found that this approach is associated with a reasonable risk of perioperative complications and can be safely applied to obese patients. Postoperative management of patients with a BMI ≥30 kg/m^2^ requires more specialized care, especially when wound dehiscence occurs.

## Competing interests

The authors declare that they have no competing interests.

## Authors’ contributions

XX, HC and QZ conceived the study and participated in its design. XX, CG, HK and QZ carried out the perioperative management of all patients, participated in the operations and contributed to the drafting of the manuscript. JT and CJ contributed to the statistical analysis. All authors read and approved the final manuscript.
